# Commentary: Clinical Approach to the Differential Diagnosis between Behavioral Variant Frontotemporal Dementia and Primary Psychiatric Disorders

**DOI:** 10.3389/fpsyt.2016.00023

**Published:** 2016-02-24

**Authors:** Manish Kumar Jha, Elena Ivleva

**Affiliations:** ^1^Department of Psychiatry, University of Texas Southwestern Medical Center, Dallas, TX, USA

**Keywords:** frontotemporal dementia, forensic psychiatry, psychosis, aggression, measurement-based care

We welcome the recently published review by Ducharme and colleagues (1) and wish to highlight matters pertinent to aggressive behavior in their report. Aggression in psychiatric hospitals has gained widespread public attention (2). Mentally ill treatment-refractory patients who display aggressive behavior are either transferred to maximum-security forensic state hospitals (3) or sent to correctional facilities, which often struggle to keep up with the epidemic of mental illness and shortage of psychiatrists (4). Fourteen percent of patients with behavioral variant frontotemporal dementia (bvFTD) present initially to psychiatric care due to criminal behaviors (5). This highlights the importance of considering bvFTD as one of the key differential diagnosis in treatment-refractory forensic patients with progressively worsening cognitive functioning and high rates of violence as well as in patients with new-onset criminal behavior. Emerging biomarker-based reports indicate substantial overlap in cognitive dysfunction and underlying anatomical and functional brain abnormalities, such as frontotemporal gray matter loss, spanning psychotic disorders (6), and dementias (1). These findings call for novel dimensional approaches to psychiatric diagnoses and treatment such as those emphasized by the Research Domain Criteria (RDoC) initiative (7).

We have reported on our successful management of such a case from a maximum-security hospital, who presented with long history of psychosis refractory to multiple treatment interventions including clozapine and electroconvulsive therapy (8). Our treatment decisions were driven by revision of diagnosis from schizoaffective disorder to FTD, based on cognition (Repeatable Battery for the Assessment of Neuropsychological Status and Montreal Cognitive Assessment) and neuroimaging (structural magnetic resonance imaging) workup, as well as by utilizing measurement-based care (MBC) (9). The principles of MBC include routine measurements of symptoms, side effect, and tolerability along with modification of treatment regime based on these measurements, while best defined for treatment of major depression can be extended to management of other chronic mental illnesses (9). We used data collected as part of state-law mandated monitoring of restraint(s) and/or seclusion(s) (10) to serve as measurements, which guided our decision to change medications or their dose. For example, during the course of treatment and as reported previously (8), we re-evaluated the utility of medroxyprogesterone by reducing its dose and frequency, which was associated with sharp increase in aggression. Utilizing principles of MBC, we re-instated the dose of medroxyprogesterone and added sertraline, which in turn resulted in even greater reduction in aggression. As impulsivity and disinhibition are hypothesized to underlie the aggression in bvFTD patients, 37.4% of whom exhibit criminal behaviors (5), we implemented structured behavioral and environmental modification programs. These interventions led to considerable improvement in the patient’s psychosis, social behaviors, impulsivity, aggression, and insight, despite persistent cognitive symptoms.

This systematic review by Ducharme and colleagues is a valuable addition to literature offering a clear, step-wise differential diagnosis algorithm directly applicable to various clinical settings. However, lack of access to neuroimaging and expert consultation services in state hospitals and correctional facilities may hinder accurate diagnosis of bvFTD leading to under- or over-diagnosis, especially as its prevalence is 10–100 times lower than primary psychiatric disorders. This calls for system-level efforts, such as greater collaboration between academic centers and forensic mental health settings (4), as well as mechanistic research targeting pathophysiology and treatment developments of this devastating neurodegenerative syndrome.

## Author Contributions

Both authors (MJ and EI) contributed to the drafting of the manuscript and critical revision of manuscript for important intellectual content.

## Conflict of Interest Statement

The authors declare that the research was conducted in the absence of any commercial or financial relationships that could be construed as a potential conflict of interest.
